# Gendered differences in AIDS and AIDS-related cause of death among youth with secondary education in South Africa, 2009–2011

**DOI:** 10.1080/17290376.2016.1242434

**Published:** 2016-10-14

**Authors:** Nicole De Wet

**Affiliations:** ^a^ PhD, Lecturer in Demography and Population Studies Programme, University of the Witwatersrand, Johannesburg, South Africa.

**Keywords:** HIV/AIDS, gender, youth, education, probability of dying, odds ratios, VIH / SIDA, le genre, la jeunesse, l'éducation, la probabilité de mourir, les rapports de cotes

## Abstract

*Background*: The prevalence of human immunodeficiency virus (HIV)/acquired immune deficiency syndrome (AIDS) is higher among females than males in Sub-Saharan Africa. Education is associated with better health outcomes. For this and other reasons, African countries have made a concerted effort to increase youth education rates. However, in South Africa males have lower secondary education rates than females, yet females have a higher prevalence of HIV/AIDS. This study examines if a gender disparity exists in AIDS mortality rates among youth with secondary education in South Africa. *Methods*: This study uses descriptive statistics and life table techniques. A sample of 4386 deaths of youth with secondary education is used. Of this total sample, 987 deaths were among males and 340 were among females with secondary education. *Results*: This study shows that AIDS mortality is higher among females than males in South Africa. Males and females with secondary education have lower AIDS mortality than all males and females in the population, yet the rates are higher for females. Using cause-deleted life tables, the probability of youth dying from HIV/AIDS practically disappears for both males and females. Odds ratio calculations show that secondary education does not have a protective effect from AIDS mortality among male and female youth. *Conclusion*: Given the gendered difference in AIDS mortality among youth with secondary education, efforts to increase secondary education among males and further research into other factors exacerbating AIDS mortality among females with secondary education is needed in the country.

## Résumé

Contexte : La prévalence du VIH/sida est plus élevé chez les femmes que chez les hommes en Afrique subsaharienne. L'éducation est associé à de meilleurs résultats pour la santé. Pour cette raison et pour d'autres, les pays africains ont fait des efforts concertés pour accroître les taux d'éducation des jeunes. Toutefois, en Afrique du Sud les mâles ont des taux d'enseignement secondaire inférieur que chez les femmes, mais les femmes ont une prévalence plus élevée du VIH/SIDA. Cette étude examine si une disparité entre les sexes existe dans les taux de mortalité due au sida chez les jeunes à l'enseignement secondaire en Afrique du Sud. Méthodes : Cette étude utilise la table de statistiques descriptives et techniques. Un échantillon de 4 386 décès de jeunes de l'enseignement secondaire est utilisé. De l'échantillon total, 987 décès ont été chez les hommes et 340 chez les femmes ayant une éducation secondaire. Résultats : Cette étude montre que la mortalité due au SIDA est plus élevé chez les femmes que chez les hommes en Afrique du Sud. Les hommes et les femmes ayant l'enseignement secondaire inférieur ont la mortalité due au sida de tous les hommes et les femmes dans la population, mais les taux sont plus élevés pour les femmes. En utilisant les tables de survie d'une cause, la probabilité de mourir des jeunes contre le VIH/SIDA semble disparaître pour les hommes et les femmes. Rapport de cotes les calculs montrent que l'enseignement secondaire n'a pas un effet protecteur de la mortalité due au sida chez les garçons et les jeunes filles. Conclusion : étant donné la différence de genre dans la mortalité due au sida chez les jeunes à l'enseignement secondaire, les efforts visant à accroître l'éducation postsecondaire chez les hommes et de nouvelles recherches sur d'autres facteurs aggravant la mortalité due au sida chez les femmes ayant une éducation secondaire est nécessaire dans le pays.

## Introduction

The prevalence of human immunodeficiency virus (HIV)/acquired immune deficiency syndrome (AIDS) among youth in Sub-Saharan Africa is high. When young, economically active populations die from AIDS, production and development are hindered. In addition to affecting national economies, households are also affected by youth mortality due to AIDS. Loss of an income earner or a parent places financial and social strains on African households in economies where there is little government assistance. Efforts to increase access and availability to antiretroviral therapy (ART) across the continent have been made. In South Africa, programme and policy makers have attempted to expand treatment to all healthcare facilities and not restrict ART to a few accredited centres in order to reduce the severity of the disease (Colvin et al., 2010). Even before this drastic step, increased availability of ART has already decreased AIDS mortality from 35.6 deaths to 2.5 deaths per 100 person-years among those accessing early treatment in the country (Lawn, Myer, Orrell, Bekker, & Wood, 2005). Currently, and due to the expanded effort made in South Africa, AIDS mortality has declined from 45.9% of all deaths in 2007 to 31.1% in 2014 (Statistics SA, 2014b). Treatments are now widely available and accessible, and while previous research has noted that accessibility (including cost of travel to clinics and stigmatisation) hamper attempts to get all HIV positive persons in the country on treatment, more recent research has noted that therapy has had a positive effect on HIV testing in the country. Results show that ART has made people feel more hopeful for a prolonged life (Nachega et al., 2004; Phakathi, Van Rooyen, Fritz, & Richter, 2011).

Despite the improvements to survival made due to the availability and accessibility of ART, prevalence of HIV/AIDS in South Africa is high. Previous statistics show that one in eight economically active adults (15–49 years old) were HIV positive, that is a rate of about 10% (Gilbert & Walker, 2002; Statistics SA, 2014c). More recently, this has increased, with later results estimating that the prevalence rate among economically active South Africans is 16.6% (Statistics SA, 2015). Those over the age of 15 years of age, and who are employed, are the economically active persons; which constitute 41.3% of the youth in South Africa (Statistics SA, 2014d). In South Africa, an adapted definition of youth is used. The country considers persons between the age of 15 and 34 years old to be youth. This was strategically designed in the post-Apartheid era to enable previously disadvantaged population groups access to certain youth benefits, such as education (Presidency of the Republic of South Africa, 2009). Using the international classification of youth as persons aged 15–24 years old, however, 50.1% of the youth population are male and 49.9% are female (Statistics SA, 2015). Unlike the population distribution, which is only slightly skewed towards more males, the distribution of HIV prevalence in the country is higher among females. An earlier study found that the HIV prevalence among females aged 15–24 years old to be as high as 15.5%, while males of the same age have a prevalence of 4.8% (Pettifor et al., 2005). The disease, if not managed correctly, causes periods of sickness and eventual death; which disrupts employment and aggravates poverty (Harrison, Newell, Imrie, & Hoddinott, 2010). In addition, the main mode of transmission of the disease in the country is heterosexual intercourse, making HIV/AIDS a highly stigmatised disease (Skinner & Mfecane, 2004).

Much attention on youth and HIV transmission in South Africa has focused on the gender differences in the attitudes of youth towards infection and modes of transition. For example, among a group of young females engaging in transactional sex in the Western Cape it was found that the risk of HIV/AIDS is not as imperative as the risk of not having material goods (Zembe, Townsend, Thorson, & Ekström, 2013). In addition, despite young females knowing that their partners have other sexual partners and being aware of the risk of HIV transmission, fear of partner violence as a consequence for asking to use condoms is common (El-Bassel, Gilbert, Rajah, Foleno, & Frye, 2000). While for males, research has addressed issues pertaining to condom use. For example, one study found inconsistent condom use among younger males (15–19 years old), compared to older males (20–24 years old) (MacPhail & Campbell, 2001).

Among youth in the country, literature has shown that multiple sexual partnerships, low condom use, transactional sex and age-disparate relationships are the main determinants of infection (Shisana et al., 2014). However, HIV/AIDS does not only affect youth through transmission and prevalence. The presence of the disease in families and communities has also been found to be detrimental. Of particular concern are the consequences of HIV/AIDS on the social and developmental aspects of youth lives. Research has found that youth whose parents are HIV positive are more prone to psychological disorders, suffer as much stigmatisation as their parents and are more likely to discontinue schooling (Cluver, Gardner, & Operario, 2007; Coombe, 2000; Skinner & Mfecane, 2004). These consequences are attributed to poverty, illness, lack of motivation and trauma, and the need for children to care for the sick and elderly with whom they are living (Coombe, 2000).

With its high prevalence of persons living with HIV/AIDS, South Africa has in the past also experienced high rates of mortality due to AIDS (Dorrington, Bourne, Bradshaw, Laubscher, & Timæus, 2001; Hosegood, Vanneste, & Timæus, 2004). Research in the early to mid-2000s showed that 48% of adult deaths in South Africa were due to AIDS (Hosegood et al., 2004). Among youth in particular, age and sex differentials have been noted with older youth and females having higher rates of AIDS mortality (De Wet, Oluwaseyi, & Odimegwu, in press).

Poverty, power dynamics within relationships, gender and race are all determinants of HIV transmission and AIDS mortality in South Africa (Dunkle et al., 2004; Simbayi et al., 2007; Whiteside, 2002). With youth being a key demographic to the country’s development and economic growth, an aspect of AIDS mortality which has not been fully explored is the rates of death among youth with secondary education in the country. South Africa’s school enrolment levels are one of the highest on the continent, with 85% males and 75% females enrolled in school (Grant & Hallman, 2008). According to the 2011 Census, 42% of youth were students, while 60% of them had completed primary school and about 40% had completed secondary school (Statistics South Africa, 2011). In 2014, unemployment increased in the country by 3.7% from the previous year. Among the unemployed are females, which increased by 0.5% from the first to second quarter; as well as persons with secondary education, which increased by 0.2% from the first to second quarter of 2014 (Statistics SA, 2014d). Education has been found in many settings to be a protective factor against illness, disease and mortality. More educated persons have more gainful employment and can therefore afford better diet and health care, and therefore also have better knowledge on health and health care (Bertakis, Azari, Helms, Callahan, & Robbins, 2000; Celik & Hotchkiss, 2000; Desai & Alva, 1998; Schillinger, Barton, Karter, Wang, & Adler, 2006). Since more of those with secondary education are becoming unemployed, and there is a gender disparity in HIV/AIDS. Therefore, it is worth examining the sex differentials in AIDS mortality among youth with secondary education in South Africa. The objective of the study, therefore, is to examine if sex is a determinant of AIDS mortality among youth who have at least a secondary level of education in South Africa.

## Methods

Data from death notification forms (DNFs) for 2009–2011 were analysed. Death registration takes place at the Department of Home Affairs, and these deaths are then updated on the national population register (NPR) (Statistics SA, 2014c). Deaths which are registered on the NPA and those not eligible for inclusion, non- South African citizens and permanent residents not listed on the NPA. Information on these deaths are collected by Statistics South Africa for processing and dissemination (Statistics SA, 2014c). Statistics South Africa processes the DNFs for all deaths in the country, regardless of civil status. For this reason the data disseminated by the organisation exceeds that of the NPR for any given year (Statistics SA, 2014c). For the country’s population distribution and economically active population, data from the General Household Surveys from 2009 to 2011 were used. This is a nationally representative, cross- sectional survey. As per the international definition of youth, males and females between the ages of 15 and 24 years old at the time of their death were included in the study. The total study population was 4386 AIDS deaths.

The quality of death notification data is based on the completeness of the records. Statistics South Africa have noted that incomplete data (or data where more than one option was selected) was less than 1% for sex, age and province of death (Statistics South Africa, 2010). Further 25% of records were missing on population group or race and for this reason this variable has been omitted from the analysis. In addition, variables pertaining to education, pregnancy status and smoking status had a cumulative amount of about 50% where values were unknown or unspecified (Statistics South Africa, 2010). This percentage of missing cases is a limitation of this study. To control for this limitation, several years of death notification data were pooled, as opposed to using only a single year. This increased the sample size even once missing data were removed from the analysis. Since death notification records are made available annually, this study uses all death records for the years 2009, 2010 and 2011. All information on the deceased (demographics and causes of death) were pooled for all years.

### Outcome measures

The outcome of interest to this study is AIDS mortality. According to the International Classification for Causes of Death (ICD-10), the code for AIDS deaths are B20–B24. From the variable ‘broad underlying cause of death’ on the DNFs, B20–B24 was used to identify AIDS deaths among the youth population.

However, due to the reports of misclassification of AIDS deaths in the country, a second outcome variable was constructed which includes underlying causes of death related to AIDS: Tuberculosis (A15–A19), Candidiasis (B37), Cryptococcosis (B49), Toxoplasmosis (B58) and Pneumocytosis (B59). These related causes of death were added to the number of AIDS (B20–B24) deaths to create the variable ‘AIDS-related’. This variable is included in the cross-tabulation analysis to demonstrate the similarity in frequency distributions. However, in order to demonstrate the relationship between secondary education and AIDS mortality, AIDS specific underlying causes of death, coded as B20–B24 on DNFs, is used in the analysis of probability of dying (Table 4) and odds ratios (Table 5).

The study also examined both AIDS and ‘AIDS-related’ causes of death in comparison to all other causes of death. AIDS and ‘AIDS-related’ deaths were removed and all other causes (communicable, non-communicable and violence or injury causes) were grouped together to form the variable ‘other death’.

### Explanatory and control variables

The main qualifying variable used in this study is secondary education. This variable was derived from the ‘highest level of education’ entry of the deceased on the DNF. Responses that the deceased’s highest level of education was Grade 12 were coded as ‘secondary education’. Education level that was unknown or missing was dropped from the analysis.

The control variables in the study were age, sex and employment status. The latter variable is coded as 1 for ‘employed’ and 0 for ‘unemployed’. In combining age with employed status (1), the ‘economically active’ variable was created. This variable is the number of employed persons in each 5-year age group, starting from age 15.

### Statistical analysis

Both descriptive and inferential methods of data analysis have been used in this study.

In addition to percentage distributions, death rates from AIDS, ‘AIDS-related’ and ‘other death’ were calculated. The general formula for the death rate from a specific cause is:
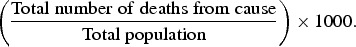


This study then used standard life table techniques. The purpose of using life table techniques is to infer the survival chances of the youth population. Most commonly, life table techniques are used to measure actual life survival probabilities by taking into account the mortality experiences of a population. However, life tables have been adapted to serve different life events such as nuptiality and fertility (Bramlett & Mosher, 2001). However, the techniques can also be used to measure the number of years expected before or probabilities that any event, whether positive (increment) or negative (decrement), will occur in a population. These techniques were selected for this study for this reason, and because life tables segregate the probabilities of mortality occurring by single ages or age groups and because sex-specific life tables can be generated, which has particular relevance to this study.

In this study associated single decrement life tables were used. Associated single decrement life tables are generated to show the probability of dying for youth from all causes, then from AIDS specifically and finally if AIDS were to be hypothetically deleted from the mortality experience of the population.

In generating the sex-specific life tables, the following steps were used:

1. The number of deaths from all causes (*_n_d_x_*) and the number of deaths from AIDS (*_n_d^i^_x_*) in each age groups needs to be generated
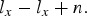
2. The probability that people will die before entering the next age group from all causes (*_n_q_x_*) and specifically that they will die from AIDS (*_n_q^i^_x_*) is created using the following equations:
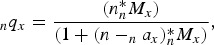

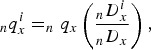

where *_n_q_x_* is the probability of dying from age *x* to *x* + *n*; *_n_D_x_* is the observed total number of deaths from all causes; and *_n_D_x_* is the observed total number of deaths from all causes.

(3) The number of survivors (*l_x_*)
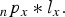
(4) The stationary population (*_n_L_x_*)
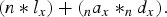
(5) The sum of years that people of a certain age are expected to live before they die (*T_x_*) is
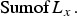
(6) Life expectancy with AIDS in the population (*e_x_*) and life expectancy if AIDS were eliminated from the population (*e*−*i_x_*) is
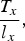

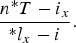

Finally, odds ratios were calculated to determine the association between secondary education and AIDS mortality by sex. For this the following formula was used:
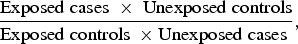


where the exposed cases are those with secondary education who died from AIDS; unexposed controls are those with none or primary education who died from ‘other causes’; exposed controls are those with none or primary education who died from AIDS and unexposed cases are those with secondary education who died from ‘other causes’.

## Results

**Fig. 1.**
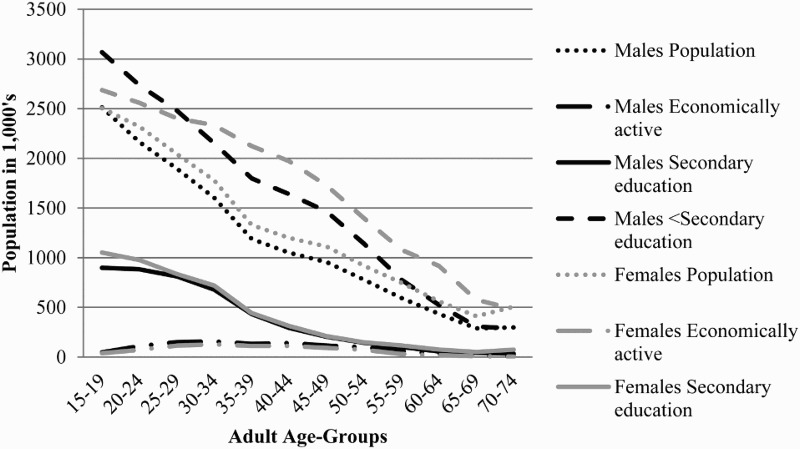
Population, economically active and education distribution (in 1000s) by age and sex in South Africa, 2009–2011.

The adult population distribution, economically active population and education status of the population by age and sex is seen in Fig. 1. In all age groups there are more people with primary or no education (less than secondary), than those with secondary education. The graph shows that among youth, more females than males have secondary education. However, only a few more males are economically active.

Having seen the population distribution of the country, what follows is to see where AIDS fits into the overall mortality experience of youth. HIV/AIDS (B20–B24) is ranked as the 11th (2.34%) leading cause of male mortality amongst youth, in the period 2009–2011 (Table 1). Male youth with secondary education have a slightly higher ranking (9th) of HIV/AIDS mortality (1.53%). For females, however, HIV/AIDS is the 4th leading cause of death at 2.83%. Unlike males, females with secondary education have a lower ranking (9th) of HIV/AIDS deaths (6.53%). In total, for youth males and females, HIV/AIDS is ranked the 5th highest cause of death. AIDS-related causes of death, such as Tuberculosis (A15–A19), is the highest ranked cause of death for females and males. Influenza and pneumonia (J09–J18) is the 4th (7.25%) leading cause of death for males, and 7th (3.11%) among young males with secondary education. While for females, influenza and pneumonia is the 4th leading cause of death among those with secondary education.

**Table 1.** AIDS and other causes of death among youth (15–24 years) by sex, South Africa, 2009–2011.

Cause of death	ICD-10 code	Males				Females				Total			
		Rank	All %	Rank	% SE	Rank	All %	Rank	% SE	Rank	All %	Rank	% SE
Intestinal infectious disease	A00–A09	5	5.31	10	1.39	4	6.52	6	5.24	4	5.9	9	3.26
*Tuberculosis*^a^	*A15–A19*	*1*	*12.99*	*5*	*6.49*	*2*	*11.43*	*1*	*18.4*	*2*	*12.22*	*2*	*12.3*
**HIV/AIDS**	**B20–B24**	**11**	**2.34**	**9**	**1.53**	**9**	**2.83**	**5**	**5.37**	**9**	**2.57**	**8**	**3.39**
*Other viral diseases*^a^	*B25–B34*							*7*	4.43			*10*	2.64
*Certain disorders involving the immune mechanism*^a^	*D80–D89*					*10*	*2.65*	*8*	*3.39*				
Diseases of the nervous system	G00–G09			8	1.56			*9*	3.24				
Other forms of heart disease	I30–I52	6	3.78			5	4.88			6	4.31		
*Influenza and Pneumonia*^a^	*J09–J18*	*4*	*7.25*	*7*	*3.11*	*3*	*8.03*	*4*	*7.93*	*3*	*7.63*	*6*	*5.45*
Ill-defined and unknown causes	R95–R99	2	11.64	6	4.51	1	13.45	3	8.58	1	12.52	4	6.49
Transport accidents	V01–V99			4	6.96			2	11.53			7	4.97
External causes	W00–X59	3	7.35	1	39.73					5	4.95	1	26.00
Assault	X85–Y09			2	11.65							3	6.65
Events of undetermined intent	Y10–Y34	8	2.85	3	9.51			10	2.93			5	6.32

Note: SE, secondary education.

^a^Denotes causes of death that could be AIDS-related.

The column percentage distribution of deaths by cause, age, sex and secondary education is seen in Table 2. Females aged 20–24 years old have consistently higher mortality, with and without secondary education, across all causes and AIDS deaths. Almost 80% of female youth deaths from AIDS were among 20–24 year olds, and for females with secondary education this increased to 85.32% of AIDS deaths in this age group. For males, about 75% of AIDS deaths were among 20–24 year olds, while males with secondary education who died from AIDS contributed almost 95% of youth male deaths during the period 2009–2011.

**Table 2.** Cause of death and secondary education by age and sex distribution (column percentages), South Africa, 2009–2011.

Age group	All causes		Secondary education		AIDS		AIDS secondary education		AIDS-related		AIDS-related secondary education	
	Male	Female	Male	Female	Male	Female	Male	Female	Male	Female	Male	Female
15–19	47.06	37.20	21.76	22.05	25.52	20.99	5.48	14.68	28.77	25.03	14.89	12.78
20–24	52.94	62.80	78.24	77.95	74.48	79.01	94.52	85.32	71.23	74.97	85.11	87.22
Total	100	100	100	100	100	100	100	100	100	100	100	100

Death rates for persons with less than and secondary education (per 1000 youth) can be seen in Table 3. The rates of death are higher for those with secondary education compared to those without. While males with less than secondary education have rates of all-cause mortality (5.87) than females (5.48), the rates for AIDS and AIDS-related causes of death are consistently higher among females. Similarly, males with secondary education have lower all-cause mortality (10.88), than females of the same education status (11.12). In addition, females with secondary education have higher rates of AIDS (0.67) and AIDS-related (0.38) deaths, than males with the same education. These rates provide a more refined depiction of gendered AIDS mortality in South Africa, since it shows rates in relation to the size of the youth population.

**Table 3.** Death rates of persons with secondary education by cause of death (COD) and sex, per 1000 youth (15–24 years) population, South Africa, 2009–2011.

Overall rate of deaths* (per 1000 youths)						
	Less than secondary education			Secondary education		
COD	Males	Females	Total	Males	Females	Total
All causes	5.87	5.48	5.67	10.88	11.12	11.00
AIDS	0.10	0.31	0.21	0.20	0.67	0.43
AIDS-related	0.54	1.44	0.99	0.12	0.38	0.25

*p < .05.

Probability of dying (*_n_q_x_*) is higher for females aged 20–24 years old and males aged 15–19 years old. The probability of dying from AIDS (*_n_q^i^_x_*) for those with less than secondary education is higher for females at both 15–19 years old (0.0008 or 0.8%) and 20–24 years old (0.004 or 4.00%), than for males at both ages (0.0003 and 0.0011). The probability of dying of AIDS were eradicated from the population (*_n_q−i_x_*) for youth with less than secondary education, and this is same for males and females aged 15–19 years old (0.0000). However, this is higher for older females (20–24 years old) at 0.0003 or 0.3%. With secondary education, the probability of dying from AIDS (*_n_q^i^_x_*) is higher for females at both ages (0.0029 or 2.9% and 0.0032 or 3.2%), than males at 1.4% and 2.7% respectively. Finally, if AIDS were eradicated from the mortality experience of youth with secondary education (*_n_q−i_x_*), the probability of dying is still higher among females (0.0001 and 0.0002), but lower than with AIDS in the population (Table 4).

**Table 4.** Probability of dying (*_n_q_x_*), probability of dying from AIDS (*_n_q^i^_x_*) and probability of dying if AIDS were eliminated in the population (*_n_q^−i^_x_*) by education, age group and sex, South Africa, 2009–2011.

	Males					Females				
Age group	Less than secondary education			Secondary education		Less than secondary education			Secondary education	
	_n_q_x_*	_n_q^i^_x_*	_n_q^−i^_x_*	_n_q^i^_x_*	_n_q^−i^_x_*	_n_q_x_*	_n_q^i^_x_*	_n_q^−i^_x_*	_n_q^i^_x_*	_n_q^−i^_x_*
15–19	0.0244	0.0003	0.0000	*0.0014*	*0.0000*	0.0218	0.0008	0.0000	*0.0029*	*0.0001*
20–24	0.0534	0.0011	0.0001	*0.0027*	*0.0001*	0.0599	0.0041	0.0003	*0.0031*	*0.0002*

*p < .05.

In Table 5, the distribution of AIDS (B20–B24 only) and all other causes of death (all other causes) are shown by secondary or none/primary education and sex for youth in South Africa from 2009 to 2011. The table also shows the results of the odds ratio calculation by sex. Males and females have a positive association with AIDS mortality if they have secondary education, compared to if they have no or primary education (odds ratio > 1). That is, exposure to secondary education is associated with AIDS mortality among youth in South Africa.

**Table 5.** Number, percentage and odds ratio of AIDS mortality by highest level of education and sex, South Africa, 2009–2011.

Highest level of education	Males			Females		
	AIDS death (B20–B24)	Other death	Total	AIDS death (B20–B24)	Other death	Total
Secondary education	974 (67.26)	54,845 (63.18)	55,819 (63.24)	3407 (67.94)	58,829 (62.46)	62,236 (62.74)
Less than secondary education	474 (32.73)	31,962 (36.82)	32,436 (36.75)	1607 (32.06)	35,357 (37.54)	36,964 (37.26)
Total	1448 (100)	86,807 (100)	88,255 (100)	5014 (100)	94,186 (100)	99,200 (100)
**Odds ratio**	**1.1975**			**1.2742**		

## Discussion

The aim of this paper was to see if there is a gender differential in AIDS mortality among youth with secondary education. Gender differentials do exist. Secondary education rates, as well as AIDS mortality among those with secondary education, are higher among females than males in South Africa. Reasons for this gendered differential include both social (low socioeconomic status leading to more risky behaviours, violence against women and gender inequality) and biological explanations for females having higher rates of infection than their male counterparts globally (WHO, 2009). The noted social issues regarding education rates in South Africa show that males have lower completion rates than females, at 47% compared to 53% (Statistics SA, 2014a). In addition, research has shown that females are more likely to test for HIV than males (Kalichman & Simbayi, 2003). While this early detection of the disease usually leads to improved health behaviour, such as early adoption of ART, socioeconomic challenges prevent this from being a reality (Lawn et al., 2005; Rosen, Fox, Gill, & Sepulveda-Amor, 2007). In South Africa, research has shown that the number of people eligible for ART dropped from 79% to 52% after the CD4 count criteria changed in 2003 (Evans, 2013). This suggests that secondary education is not a sufficient preventative measure against AIDS mortality.

Females in this age group have a lower probability of survival, with and without AIDS as a cause of death, compared to males. These are females who are also reproductively active and therefore the competing cause of death is maternal mortality. In South Africa, maternal mortality for females is between 230 and 575 maternal deaths per 100,000 live births (Pattinson, 2008). This could explain the higher probability of dying in this age group, compared to males, even without the presence of HIV/AIDS in the population. This is again coupled with the social challenges females in South Africa face, which other studies have attested to. Research in South Africa has found that young female migrants have higher odds of AIDS mortality than their male counterparts (Clark, Collinson, Kahn, Drullinger, & Tollman, 2007). Females, in particular those who are poor, marginalised or uneducated have limited access to healthcare resources, relating to more adverse health outcomes (Chauke, Munzhelele, & Maiwashe, 2015). Also females who suffer higher rates of unemployment, are also both affected and infected by HIV/AIDS and also assume the role of primary caregivers to children and the elderly (Chauke et al., 2015; Mncwango & Luvuno, 2015; Wright, Neves, Ntshongwana, & Noble, 2015). Within this context of numerous social plights, their individual health outcomes suffer and this explains the higher probability of dying over males in the population.

The classification of AIDS deaths in South Africa has notably improved. Previous research found that as many as 94% of cause of death data from 1996 to 2006 was misclassified (Birnbaum, Murray, & Lozano, 2011). Since then data improvement efforts by Statistics South Africa have ensured a better and more complete classification of the stigmatised disease (Statistics SA, 2010). As a result, the data used in this paper is more complete, but frequencies among youth, especially younger youth (15–19 years old) are low and hence the need to pool the data. In addition, Anderson and Phillips (2006) have suggested that there are several infectious diseases which only occur to persons with a vulnerable immune system, which could be attributed to the presence of the HI-Virus. These include Candidiasis, Cryptococcosis, Pneumocytosis and Toxoplasmosis (Anderson & Phillips, 2006). Further, research has acknowledged that Tuberculosis and AIDS are relatable, and that many AIDS deaths in South Africa may have been misclassified as Tuberculosis (Anderson & Phillips, 2006; Groenewald, Nannan, Bourne, Laubscher, & Bradshaw, 2005). Tuberculosis and AIDS have similar disease symptoms and are therefore easy to misdiagnose. Also in high HIV epidemic regions, Tuberculosis is prevalent, making the diseases indistinguishable in the absence of thorough post-mortem examinations (Mukadi, Maher, & Harries, 2001). Further, rates of co-infection of Tuberculosis and HIV in South Africa are high, research shows that these rates are as high as 755 cases per 100,000 pregnant women (Pillay et al., 2001). For this reason, the study included these infectious diseases to create a more reliable depiction of AIDS mortality in the country. Further, since this study used secondary data, which is freely accessible from the Statistics South Africa website, there was no need to obtain ethical clearance.

In conclusion, the benefit to survival if AIDS as a cause of death were eliminated is clear. Therefore efforts to reduce AIDS mortality in the country need to be strengthened. Efforts aimed at youth in particular are important, since this sub-population constitute the majority and are beneficial to South Africa’s future and sustainable development. In order to address and strengthen programmes and interventions however, the pathways through which secondary education acts as a prohibiting factor for female survival needs to be examined. Research on the specific mechanisms through which education, employment and other socioeconomic factors are associated with AIDS mortality needs to be conducted. For female youth, the quality of education and related knowledge of HIV infection and spread need to be assessed. It is possible that the education level of females is not the problem aggravating AIDS mortality, but rather the socioeconomic status of females in the country. There remains a need for continued investigation into this epidemic in order to fully understand and address the many complex ways in which AIDS continues to affect youth in all populations.
